# Assessing the Effect of Hypothyroidism on Maternal Morbidity and Pregnancy Outcome Among Pregnant Women Attending a Tertiary Care Hospital in Central India: A Prospective Study

**DOI:** 10.7759/cureus.104741

**Published:** 2026-03-05

**Authors:** Kirti Shrivastava, Bishwajeet Thakur, Amit Agrawal, Priyanka Diwan, Avadesh Diwakar, Kalpana Arya

**Affiliations:** 1 Community Medicine, Gajra Raja Medical College, Gwalior, Gwalior, IND; 2 Community Medicine, Atal Bihari Vajpayee Government Medical College, Vidisha, Vidisha, IND; 3 Community Medicine, Government Medical College, Datia, Datia, IND

**Keywords:** fetal outcome, hypothyroidism, india, maternal morbidity, pregnancy

## Abstract

Hypothyroidism is among the most prevalent endocrine disorders complicating pregnancy, leading to a broad range of adverse maternal and neonatal outcomes, especially when thyroid function is inadequately managed. Despite the significant disease burden in India, regional evidence on the impact of uncontrolled hypothyroidism on fetomaternal outcomes remains scarce. This prospective observational study evaluated the socio-demographic profile, maternal morbidities, neonatal outcomes, and influence of thyroid control status on pregnancy outcomes among hypothyroid pregnant women at a tertiary care center in Central India. Conducted in the Department of Obstetrics and Gynecology at Jaya Arogya (J.A.) Group of Hospitals, Gwalior, from May 2023 to April 2024, the study included 215 pregnant women diagnosed with hypothyroidism. Data on socio-demographic characteristics, obstetric history, treatment status, maternal complications, and neonatal outcomes were collected using a pretested semi-structured proforma. Participants were categorised into controlled and uncontrolled groups based on thyroid function status. Data were analyzed using IBM SPSS Statistics for Windows, V. 25.0 (IBM Corp., Armonk, NY, USA), with categorical variables expressed as frequencies and percentages and associations assessed using chi-squared tests. The mean age of participants was 26.62 ± 5.09 years. The most common maternal complications were anaemia (48%), postpartum haemorrhage (28%), and intrauterine growth restriction (IUGR) (24.6%), while low birth weight (43.7%), preterm birth (19.1%), and neonatal intensive care unit (NICU) admission (16.8%) were the predominant neonatal adverse outcomes. Uncontrolled hypothyroidism was significantly associated with higher rates of anaemia, IUGR, gestational diabetes mellitus, oligohydramnios, preterm birth, low birth weight, NICU admission, and postpartum haemorrhage (p < 0.05 for all). These findings highlight that uncontrolled hypothyroidism significantly increases maternal and neonatal morbidity, emphasizing the need for early detection, regular monitoring, and optimal treatment to improve fetomaternal outcomes, particularly in resource-limited settings.

## Introduction

Thyroid dysfunction is a common endocrine abnormality in pregnancy and increasingly contributes to adverse maternal and fetal outcomes. Hypothyroidism, including overt and subclinical hypothyroidism, isolated hypothyroxinemia, and autoimmune thyroid disease, affects a substantial proportion of pregnant women worldwide and poses a major public health concern because of its impact on pregnancy outcomes [1,2].

Population- and hospital-based studies report a high prevalence of thyroid dysfunction during pregnancy. Mahadik et al. observed thyroid disorders in 11% of pregnant women, with subclinical hypothyroidism as the most frequent presentation [2]. In North India, Dhabhai et al. reported hypothyroidism in 29.2% of women in early pregnancy, highlighting the substantial disease burden in low- and middle-income countries [3]. These findings emphasise the need for early screening and prompt identification of thyroid dysfunction during pregnancy.

Studies have consistently linked maternal hypothyroidism to adverse maternal and fetal outcomes. Research shows increased risks of anaemia, preeclampsia, cesarean delivery, and postpartum complications among hypothyroid pregnant women [2]. Fetal consequences include low birth weight, preterm birth, low Apgar scores, higher neonatal intensive care unit (NICU) admission rates, and congenital anomalies [2,4]. Kiran et al. demonstrated that the timing of diagnosis critically influences fetal outcomes, with early-onset disease significantly increasing the risk of low birth weight and congenital anomalies, particularly cardiovascular defects [4].

Beyond overt and subclinical hypothyroidism, however, emerging evidence shows that autoimmune thyroid disease and isolated hypothyroxinemia independently affect pregnancy outcomes. Kiran et al. reported thyroid antibody positivity in 74.7% of hypothyroid pregnant women and found significant associations with pre-gestational diabetes and fetal respiratory outcomes [1]. Similarly, isolated hypothyroxinemia increases the risk of preterm birth, premature rupture of membranes, gestational diabetes, macrosomia, and fetal distress [5,6].

Levothyroxine therapy demonstrates protective effects against adverse outcomes. A systematic review and meta-analysis by Ding et al. showed that levothyroxine treatment significantly reduces pregnancy loss, preterm delivery, and gestational hypertension in women with subclinical hypothyroidism [7]. These findings support the importance of early screening and timely treatment to prevent complications.

Despite substantial literature linking hypothyroidism in pregnancy with adverse fetomaternal outcomes, limited prospective data from Central India have specifically evaluated whether biochemical thyroid control status during pregnancy modifies these risks. Most available Indian studies describe prevalence or overall outcome patterns without distinguishing between adequately controlled and persistently uncontrolled hypothyroid states.

Thyroid hormone deficiency may impair placental angiogenesis, trophoblastic invasion, and uteroplacental perfusion, thereby predisposing to fetal growth restriction, anaemia, and preterm birth. However, the extent to which biochemical control mitigates these risks in routine tertiary care settings remains underexplored. The objective of this study is to assess the association between thyroid control status (controlled vs uncontrolled hypothyroidism) and maternal and neonatal outcomes and describe the socio-demographic and clinical profile of hypothyroid pregnant women attending a tertiary care hospital.

## Materials and methods

Study design and setting

A prospective observational study was conducted in the Department of Obstetrics and Gynecology, Jaya Arogya (J.A.) Group of Hospitals, a tertiary care teaching hospital affiliated with Gajra Raja Medical College, Gwalior, Madhya Pradesh, India.

Study duration

The study was carried out over a period of one year, from May 2023 to April 2024.

Study population

The study included both previously diagnosed hypothyroid women on levothyroxine therapy prior to conception and women newly diagnosed during pregnancy based on trimester-specific thyroid-stimulating hormone (TSH) levels. Among previously diagnosed cases, the duration of hypothyroidism ranged from six months to eight years.

Sample size and sampling technique

Sample size was calculated using the single-proportion formula: $\mathrm{n}=\mathrm{Z}^{2}\times\mathrm{p}\left(1&minus;\mathrm{p}\right)/\mathrm{d}^{2}$. Here, Z = 1.96 (95% confidence level), p = 0.276 (prevalence of preterm birth among hypothyroid pregnancies) [8], and d = 0.06 (absolute precision).

The minimum calculated sample was 214. After adding 10% for possible loss to follow-up, the final target sample was 240. Participants were recruited using purposive consecutive sampling until the desired sample size was achieved.

Inclusion criteria

Pregnant women aged 18 years or older with a confirmed diagnosis of overt or subclinical hypothyroidism, defined as TSH levels above the trimester-specific reference range, who were receiving follow-up care at the antenatal outpatient department and provided written informed consent, were eligible for inclusion in the study.

Exclusion criteria

Women with pre-existing diabetes mellitus, chronic hypertension, renal or hepatic disorders, or systemic autoimmune diseases, multiple gestations, known fetal congenital anomalies at baseline, a history of thyroid surgery or radioactive iodine therapy, or incomplete clinical or outcome data were excluded from the study.

Data collection tools and procedure

Data were collected using a predesigned, pretested semi-structured proforma. Information regarding socio-demographic characteristics, obstetric history, treatment status, and clinical parameters was recorded. All participants received levothyroxine therapy. Initial dosing ranged from 25 to 100 mcg/day depending on baseline TSH levels and body weight. Dose titration was performed at 4-6-week intervals based on repeat TSH measurements.

Thyroid function was assessed using trimester-specific reference ranges for serum TSH as recommended by the American Thyroid Association guidelines (first trimester: 0.1-2.5 mIU/L; second trimester: 0.2-3.0 mIU/L; third trimester: 0.3-3.0 mIU/L) [9]. Classification as "uncontrolled hypothyroidism" required at least two consecutive TSH readings above trimester-specific reference ranges despite dose adjustment. Medication adherence was assessed through self-report and prescription review during antenatal visits.

Maternal morbidities such as anaemia, gestational diabetes mellitus (GDM), pregnancy-induced hypertension, preeclampsia, oligohydramnios, postpartum haemorrhage (PPH), and intrauterine growth restriction (IUGR) were documented. Fetal outcomes, including preterm birth, low birth weight, NICU admission, and intrauterine fetal demise, were recorded at the time of delivery.

Anthropometric and socioeconomic assessment

Anthropometric measurements were taken using standard methods. Body mass index (BMI) was calculated as weight in kilograms divided by height in meters squared (kg/m²) and classified according to the World Health Organization criteria [10]. Socioeconomic status was assessed using the modified BG Prasad scale, updated with the consumer price index for January 2025, to reflect the current economic classification [11].

Operational definitions

Anaemia was defined as a haemoglobin concentration <11 g/dL at any time during pregnancy, according to the World Health Organization criteria for anaemia in pregnant women [12]. GDM was diagnosed using the Diabetes in Pregnancy Study Group of India (DIPSI) criteria, defined as a two-hour plasma glucose value ≥140 mg/dL following a 75 g oral glucose load irrespective of fasting status [13]. IUGR was defined as estimated fetal weight below the 10th percentile for gestational age based on standardised fetal growth charts and obstetric practice guidelines [14]. Preterm birth was defined as delivery occurring before 37 completed weeks of gestation as per the World Health Organization definition [15]. PPH was defined as cumulative blood loss ≥500 mL after vaginal delivery or ≥1000 mL after cesarean section in accordance with the American College of Obstetricians and Gynecologists guidelines [16]. NICU admission was defined as neonatal admission exceeding 24 hours for medical indication requiring specialised monitoring or intervention as per standard neonatal care recommendations [17].

Ethical considerations

Ethical clearance (approval number: 94/IEC-GRMC/2023) was obtained from the Institutional Ethics Committee of Gajra Raja Medical College, Gwalior, before the initiation of the study. Written informed consent was obtained from all participants. Confidentiality and anonymity of participants were strictly maintained throughout the study.

Statistical analysis

Data were entered in Microsoft Excel 2019 (Microsoft Corporation, Redmond, WA, USA) and analysed using IBM SPSS Statistics for Windows, V. 25.0 (IBM Corp., Armonk, NY, USA). Categorical variables were expressed as frequency and percentage. Associations between thyroid control status and outcomes were assessed using the chi-squared or Fisher's exact test. Crude odds ratios (OR) with 95% confidence intervals (CI) were calculated. Multivariable logistic regression analysis was performed to adjust for potential confounders including maternal age, BMI, gravidity, socioeconomic status, and booking status. Adjusted OR (aOR) with 95% CI were reported. A p-value of <0.05 was considered statistically significant.

## Results

The study included 215 hypothyroid pregnant women in the final analysis. Most participants were aged 18-25 years (62.3%), followed by 25-35 years (32.6%); the mean age was 26.62 ± 5.09 years. Most were Hindu (92.6%) and nearly equally distributed between rural (49.3%) and urban (50.7%) residences. A large proportion lived in joint families (70.2%) and belonged to the upper-middle socioeconomic class (41.4%). Nearly half had completed high school education (45.6%), and most were homemakers (89.3%). A normal BMI (18.5-24.9 kg/m²) was observed in 43.3%, while 42.8% were overweight. Most women were booked for antenatal care (73%), used iodised salt (99.1%), and were fully immunised (74.4%). Multigravida status was present in 53%, and 46% reported a history of infertility. Of the 215 participants, 92 (42.8%) were enrolled in the first trimester, 78 (36.3%) in the second trimester, and 45 (20.9%) in the third trimester (Table 1).

**Table 1:** Socio-demographic and baseline characteristics of hypothyroid pregnant women (n = 215) Values are expressed as number (percentage). BMI: body mass index

Variable	Category	n (%)
Age (years)	18-25	134 (62.3)
	25-35	70 (32.6)
	35-45	11 (5.1)
Religion	Hindu	199 (92.6)
Residence	Rural	106 (49.3)
	Urban	109 (50.7)
Family type	Joint	151 (70.2)
Socioeconomic status	Class II (upper-middle)	89 (41.4)
Education	High school	98 (45.6)
Occupation	Homemaker	192 (89.3)
BMI (kg/m²)	18-24.9	93 (43.3)
	25-29.9	92 (42.8)
Booked status	Booked	157 (73)
Iodised salt use	Yes	213 (99.1)
Immunisation status	Complete	160 (74.4)
Gravidity	Multigravida	114 (53)
History of infertility	Present	99 (46)

Anaemia was the most frequent complication (48.8%), followed by PPH (27.9%) and IUGR (24.6%). GDM occurred in 14%, oligohydramnios in 11.2%, gestational hypertension in 9.8%, and preeclampsia in 5.1%. Cesarean delivery was performed in 40.9% of cases (Table 2).

**Table 2:** Maternal morbidity observed among hypothyroid pregnant women (n = 215) Frequency distribution of maternal morbidities among hypothyroid pregnancies (n = 215). Values are expressed as number (percentage). IUGR: intrauterine growth restriction; PPH: postpartum haemorrhage; GDM: gestational diabetes mellitus; LSCS: lower segment cesarean section

Maternal morbidity	n (%)
Anaemia	105 (48.8)
IUGR	53 (24.6)
PPH	60 (27.9)
GDM	30 (14)
Oligohydramnios	24 (11.2)
Gestational hypertension	21 (9.8)
Preeclampsia	11 (5.1)
LSCS	88 (40.9)

Low birth weight emerged as the most prevalent adverse outcome, affecting 94 (43.7%) infants. Preterm birth was noted in 41 (19.1%) cases, while 36 (16.7%) neonates required admission to the NICU. Intrauterine fetal demise occurred in three (1.4%) pregnancies, and congenital anomalies were rare, observed in just one (0.5%) case (Table 3).

**Table 3:** Fetal outcome among babies born to hypothyroid mothers (n = 215) Values are represented as frequency and percentage out of the total. NICU: neonatal intensive care unit; IUFD: intrauterine fetal demise

Fetal outcome	n (%)
Preterm birth	41 (19.1)
Low birth weight	94 (43.7)
NICU admission	36 (16.7)
IUFD	3 (1.4)
Congenital anomaly	1 (0.5)

Uncontrolled hypothyroidism was associated with higher odds of maternal anaemia (OR: 3.46; 95% CI: 1.95-6.12), IUGR (OR: 3.81; 95% CI: 1.97-7.37), preterm birth (OR: 3.09; 95% CI: 1.49-6.39), low birth weight (OR: 3.80; 95% CI: 2.17-6.64), and PPH (OR: 3.39; 95% CI: 1.83-6.27). GDM (χ² = 5.38; p = 0.020) and oligohydramnios (χ² = 4.01; p = 0.040) were also significantly more frequent in the uncontrolled group. Among neonatal outcomes, preterm birth (χ² = 10.12; p = 0.001), low birth weight (χ² = 21.74; p < 0.001), and admission to the NICU (χ² = 9.58; p = 0.002) were statistically significantly associated with poor thyroid control. PPH similarly demonstrated a significant difference between groups (χ² = 14.09; p < 0.001) (Table 4).

**Table 4:** Comparison of maternal morbidity and fetal outcome between controlled and uncontrolled hypothyroid pregnancies (n = 215) Controlled: trimester-specific TSH within reference range on treatment; uncontrolled: persistently deranged TSH during pregnancy. Values are represented as frequency and percentage out of the total. The chi-squared (χ^2^) test was used as the test of association. A p-value of less than 0.05 was considered statistically significant. *p<0.05; **p<0.01; ***p<0.001. NICU: neonatal intensive care unit; df: degrees of freedom; TSH: thyroid-stimulating hormone

Outcome	Controlled (n = 121) n (%)	Uncontrolled (n = 94) n (%)	Crude OR (95% CI)	P-value
Maternal outcomes				
Anaemia	42 (34.7%)	61 (64.9%)	3.46 (1.95-6.12)	<0.001***
Gestational diabetes mellitus	11 (9.1%)	19 (20.2%)	2.52 (1.14-5.55)	0.020*
Oligohydramnios	9 (7.4%)	15 (15.9%)	2.35 (0.98-5.62)	0.040*
Postpartum haemorrhage	21 (17.4%)	39 (41.5%)	3.39 (1.83-6.27)	<0.001***
Lower segment cesarean section	45 (37.2%)	43 (45.7%)	1.42 (0.84-2.41)	0.19 NS
Neonatal outcomes				
Intrauterine growth restriction	17 (14%)	36 (38.3%)	3.81 (1.97-7.37)	<0.001***
Preterm birth	14 (11.6%)	27 (28.7%)	3.09 (1.49-6.39)	0.001**
Low birth weight	36 (29.7%)	58 (61.7%)	3.80 (2.17-6.64)	<0.001***
NICU admission	12 (9.9%)	24 (25.5%)	3.13 (1.46-6.73)	0.002**

Cesarean section rates were higher in the uncontrolled group compared to the controlled group; however, the difference did not reach statistical significance after adjustment for confounders.

## Discussion

The present study highlights a significant burden of maternal and fetal morbidity among pregnant women with hypothyroidism, particularly those with uncontrolled thyroid levels. High incidences of anaemia, IUGR, PPH, low birth weight, and preterm birth indicate that inadequate thyroid control was associated with higher rates of adverse obstetric and fetal outcomes in this setting. These findings suggest that effective biochemical control, rather than mere diagnosis, was associated with lower observed complication rates and more favourable pregnancy outcomes. Early detection, appropriate levothyroxine titration, and regular monitoring may contribute to improved outcomes in routine antenatal care.

Global and mechanistic evidence support these observations. Mallawa Kankanamalage et al. described how gestational hypothyroidism disrupts placental angiogenesis, trophoblastic invasion, and nutrient transfer, leading to placental insufficiency, fetal growth restriction, pregnancy loss, and neurodevelopmental impairment [18]. Large quantitative syntheses further confirm these associations. Han et al. reported significantly increased odds of preterm birth, gestational diabetes, and fetal distress among women with isolated hypothyroxinemia [5]. Nazarpour et al. similarly documented higher rates of adverse obstetric outcomes even in mild or subclinical disease [6]. These pooled analyses demonstrate that subtle thyroid hormone deficiencies were associated with clinically meaningful complications, aligning with the adverse outcomes observed in our cohort.

Thyroid autoimmunity also independently contributes to poor outcomes. Kiran et al. showed that thyroid antibody positivity was associated with higher rates of adverse maternal and fetal events, including neonatal morbidity, even when thyroid hormone levels appeared controlled [1]. In a related analysis, Kiran et al. further reported higher rates of fetal complications and congenital anomalies in pregnancies complicated by hypothyroidism [4]. These findings suggest that immunological mechanisms, in addition to hormonal deficiency, may impair placental function. Since our study did not assess antibody status, undetected autoimmunity may partially explain the higher neonatal complications observed.

Evidence regarding levothyroxine therapy remains heterogeneous but largely supports early and adequate treatment. Ding et al. demonstrated that levothyroxine therapy was associated with reduced risks of pregnancy loss, preterm birth, and gestational hypertension among women with subclinical hypothyroidism [7]. Conversely, Jiao et al. found limited or statistically nonsignificant benefits, attributing these findings to low-quality evidence, delayed initiation, and inadequate statistical power [19]. However, more recent and methodologically robust meta-analyses by Bein et al. and Sankoda et al. reported that levothyroxine treatment was associated with lower rates of miscarriage, fetal death, and preterm birth, particularly in women with higher baseline TSH concentrations [20,21]. Collectively, these data indicate that treatment effectiveness may depend on disease severity and timely initiation. In our study, adequate biochemical control was associated with lower observed complication rates compared to persistently uncontrolled thyroid status.

Indian studies reveal similar trends and offer strong contextual relevance. Mahadik et al. identified higher rates of anaemia, low birth weight, NICU admission, and preeclampsia among hypothyroid pregnant women, closely reflecting the complication profile observed in our population [2]. In a large population-based cohort, Dhabhai et al. demonstrated that adequately treated hypothyroid women achieved outcomes comparable to euthyroid pregnancies, highlighting that proper management was associated with more favourable outcomes [3]. Similarly, Mavatkar et al. documented a high prevalence of subclinical hypothyroidism and reported adverse fetomaternal outcomes when treatment was delayed or inadequate [8]. These studies emphasise the importance of early diagnosis and strict adherence to therapy, consistent with our observations.

Anaemia prevalence remains high in the Indian population due to nutritional deficiencies, which may independently influence outcomes such as IUGR and PPH. Similarly, overweight status and multigravidity may contribute to gestational diabetes risk. Although multivariable adjustment was performed, residual confounding cannot be excluded.

Guideline-based evidence further supports routine screening and management. The American Thyroid Association recommends trimester-specific TSH targets, early testing, and prompt levothyroxine therapy as part of evidence-based care [9]. From a public health perspective, background maternal health factors also influence vulnerability. Chowdhury et al. reported a high prevalence of preconception morbidity, including thyroid dysfunction and anaemia, among Indian women of reproductive age [22], which may amplify risks during pregnancy. Lakiang et al. highlighted gaps in screening and advocated universal antenatal screening for non-communicable diseases, including thyroid disorders, to prevent missed diagnoses and avoidable complications in India [23]. Overall, the present study aligns closely with both international and Indian evidence.

While prior studies established associations between hypothyroidism and adverse outcomes, our findings demonstrate that uncontrolled thyroid status was associated with higher rates of maternal and fetal morbidity within a real-world tertiary care setting. Adequate biochemical control was associated with lower observed complication rates. These findings support strengthening early antenatal thyroid evaluation, adherence to guideline-based management, and close biochemical monitoring, which may contribute to improved fetomaternal outcomes in high-prevalence regions such as Central India.

Limitations

This study has several limitations. First, because this is a single-centre tertiary care study with purposive consecutive sampling, referral bias and limited generalizability are possible. Second, the absence of a euthyroid comparison group restricts causal inference. Third, despite multivariable adjustment, residual confounding may persist. Fourth, thyroid antibody status and iodine levels were not assessed. Fifth, classification based on TSH measurements may introduce misclassification bias due to physiological variability. Finally, the study was powered for prevalence estimation and may be underpowered for subgroup comparisons.

Future direction of the study

To enhance generalizability and minimise referral bias, future research should include multicentric studies with larger, more diverse populations. Longitudinal follow-up of mothers and infants is essential to evaluate the long-term neurodevelopmental, growth, and cognitive outcomes linked to maternal hypothyroidism. Studies that incorporate trimester-specific thyroid parameters and thyroid antibody status can more effectively stratify risk and guide targeted therapy. Randomised trials should assess the optimal levothyroxine dosing, timing of initiation, and monitoring protocols. Furthermore, research on the implementation and cost-effectiveness of universal antenatal thyroid screening, along with community-based education and preconception care strategies, will help strengthen preventive programs and inform evidence-based maternal health policies.

## Conclusions

Uncontrolled hypothyroidism during pregnancy was associated with higher rates of maternal anaemia, IUGR, preterm birth, low birth weight, PPH, and NICU admission. Adequate biochemical control was associated with comparatively lower complication rates. These findings support the importance of early antenatal thyroid evaluation, close monitoring, and guideline-based management to optimise fetomaternal outcomes. However, causal inference cannot be established from this observational study.
